# Telemedicine and Digital Tools in Dentistry: Enhancing Diagnosis and Remote Patient Care

**DOI:** 10.3390/medicina61050826

**Published:** 2025-04-30

**Authors:** Amelia Surdu, Cezar Ilie Foia, Ionut Luchian, Daniela Trifan, Dana Gabriela Budala, Mihaela Monica Scutariu, Corina Ciupilan, Bogdan Puha, Diana Tatarciuc

**Affiliations:** 1 Department of Oral Diagnosis, Faculty of Dental Medicine, “Grigore T. Popa” University of Medicine and Pharmacy, 700115 Iasi, Romania; 2 Faculty of Dental Medicine, “Grigore T. Popa” University of Medicine and Pharmacy, 700115 Iasi, Romania; 3 Department of Periodontology, Faculty of Dental Medicine, “Grigore T. Popa” University of Medicine and Pharmacy, 700115 Iasi, Romania; 4 Department of Orthodontics, Faculty of Dental Medicine, “Nicolae Testemitanu” University of Medicine and Pharmacy, MD-2004 Chisinau, Moldova; 5 Department of Dentures, Faculty of Dental Medicine, “Grigore T. Popa” University of Medicine and Pharmacy, 700115 Iasi, Romania; 6 Department of Morpho-Functional Science, Faculty of Medicine, “Grigore T. Popa” University of Medicine and Pharmacy, 700115 Iasi, Romania; 7 Department of Orthopaedics and Traumatology, Faculty of Medicine, “Grigore T. Popa” University of Medicine and Pharmacy, 700115 Iasi, Romania; 8 Department of Medicine Specialties, Faculty of Medicine, “Grigore T. Popa” University of Medicine and Pharmacy, 700115 Iasi, Romania

**Keywords:** teledentistry, telemedicine, oral health, digital tools, telehealth, remote diagnosis

## Abstract

Teledentistry enhances access to oral healthcare by enabling remote consultations, diagnosis, and patient management. This paper explores its applications, benefits, challenges, and impact on modern dentistry. A comprehensive review of existing literature and case studies was conducted to examine the effectiveness of teledentistry. Key aspects analyzed include digital imaging, AI (artificial intelligence)-assisted diagnostics, and cloud-based patient records, which facilitate early disease detection, reduce wait times, and minimize unnecessary visits. The review also highlights how teledentistry improves collaboration among dental professionals for better treatment planning. Challenges include legal barriers, data security concerns, and limited digital infrastructure. Standardized protocols and professional training are essential for effective implementation. Future advancements in AI and telecommunication technologies will further integrate teledentistry into standard practice, improving accessibility and efficiency in oral healthcare.

## 1. Introduction

Technological advancements in dentistry have been substantial in the past few years. Digital diagnostic imaging services, technologies, software, and communication have all seen significant advancements in their application in analysis and post-treatment remote monitoring and evaluation [1]. The field of dentistry has come a long way in the last century, thanks to the advancements in information technology [2]. Digital platforms have increasingly been integrated into dental care delivery, complementing in-person visits by enabling remote consultations, diagnosis, and follow-up monitoring in appropriate clinical contexts.

In the context of healthcare, telemedicine is just one link in the chain. By strengthening this link, it can increase healthcare’s efficacy and quality [3]. Many other types of healthcare facilities are already making use of telemedicine, including universities, community hospitals, managed care organizations, rural hospitals, and even worldwide networks that connect hospitals in wealthy nations to those in poorer nations.

One branch of telemedicine that focuses on dental care is called teledentistry, and it handles everything from networking to digital information exchange to remote consultations to workup and analysis [4]. This innovative approach allows dentists to remotely diagnose, monitor, and consult on various oral health conditions, reducing the need for in-person visits while maintaining high standards of care. Through the use of digital platforms, patients can receive expert opinions, follow-up care, and even treatment guidance from specialists, regardless of their geographical location.

Oral healthcare is the focus of teledentistry, a subset of telehealth that also includes telemedicine. The terms teledentistry and telehealth are sometimes used interchangeably, although they really include different aspects of remote healthcare and healthcare practitioner training and education [5]. The integration of teledentistry into different dental specialties has expanded its applications significantly. Fields, such as teleperiodontology, teleorthodontics, and telepedodontics now utilize remote consultations, AI-driven diagnostics, and cloud-based patient management systems to enhance treatment accuracy and efficiency [6,7].

Similar to telemedicine, teledentistry incorporates key elements, such as tele-triage, teleconsultation, telediagnosis, telemonitoring, and tele-assistance, with each contributing to improved patient outcomes. These tools help streamline workflows, facilitate interdisciplinary collaboration, and support real-time decision-making in dental care. As technology advances, the role of teledentistry will continue to grow, offering more precise diagnostics, better preventive strategies, and enhanced patient engagement, ultimately shaping the future of modern dentistry [8,9].

In recent years, teledentistry and associated digital tools have emerged as transformative elements in the delivery of oral healthcare. These technologies include high-resolution intraoral cameras, digital radiography, electronic health records, teleconsultation platforms, and artificial intelligence-based diagnostic systems [3,5]. They serve a wide array of purposes, from remote diagnosis and treatment planning to patient education, postoperative monitoring, and interdisciplinary collaboration [4]. However, beyond emergency use, there is an increasing need to assess the structured integration of teledentistry into long-term dental practice [7,9]. While numerous narrative reviews exist, many focus on either the technological aspects or on specific subspecialties, without offering a comprehensive, up-to-date synthesis of its cross-disciplinary applications. Furthermore, gaps remain in understanding the influence of digital tools on health literacy, patient outcomes, and systemic healthcare delivery.

This study aims to bridge these gaps by analyzing the current applications of teledentistry across multiple dental disciplines and highlighting how digital tools enhance communication, diagnosis, and continuity of care. The review also discusses current limitations, ethical considerations, and future directions that may guide the implementation of more equitable and efficient teledental services

## 2. Literature Review

A comprehensive search was conducted across multiple scientific databases, including PubMed, Scopus, EMBASE, Web of Science, the Cochrane Database of Systematic Reviews, and Google Scholar. The search term included “teledentistry”, “telemedicine in dentistry”, “digital tools in oral healthcare”, “remote dental diagnosis”, and related keywords. Peer-reviewed journal articles, systematic reviews, and relevant conference papers published within the last 15 years were prioritized to ensure the inclusion of the latest technological advancements.

✓ The inclusion criteria were as follows:
  - Studies in English;
  - Articles discussing the application of telemedicine and digital tools in dental care;
  - Papers covering technological innovations in dental diagnosis and patient monitoring;
  - Research highlighting the benefits, limitations, and future trends in teledentistry.
✓ The exclusion criteria were as follows:
  - Articles focusing solely on traditional dental practices without technological integration;
  - Papers with insufficient data or non-peer-reviewed sources.
✓ Data collection and analysis

The selected articles were reviewed for information on the implementation of digital tools in dental healthcare, as well as their efficiency and their impact on patient care. The data were categorized into key themes, such as diagnostic imaging, AI-assisted dentistry, remote patient monitoring, digital communication platforms, and administrative improvements in dental practice.

Since this study is a literature review, no human subjects were directly involved. However, ethical considerations were maintained by ensuring the use of reliable and properly cited sources, avoiding plagiarism, and presenting an objective synthesis of findings.

By following this methodology, the study aims to provide a comprehensive understanding of how teledentistry and digital tools are transforming modern dentistry, highlighting their role in enhancing diagnostic accuracy, remote patient care, and overall efficiency in dental practice.

### 2.1. The Beginnings

Teledentistry traces its origins to the late 20th century as a subset of telemedicine, developed to address the challenges of providing dental care in remote and underserved areas. The concept emerged with early advancements in telecommunication, allowing healthcare professionals to exchange medical information over long distances [10].

One of the earliest recorded uses of teledentistry was in the United States military, where dental professionals explored ways to provide remote care to soldiers stationed in isolated locations [11]. In the mid-1990s, the Total Dental Access (TDA) project was launched by the U.S. Army to test the feasibility of transmitting digital dental records and radiographs to specialists for remote diagnosis and treatment planning [12]. This initiative demonstrated that electronic communication could enhance dental care by overcoming geographic barriers [12].

During the same period, universities and research institutions began investigating the store-and -forward method, where patient data, including X-rays and intraoral images, were captured and sent to specialists for analysis [13]. This method reduced the need for immediate in person evaluation and allowed for better resources allocation in dental healthcare [14]. This early development laid the foundation for the modern evolution of teledentistry, proving that digital communication could support remote dental care and improve patient access to specialized treatments [15,16].

By the late 1990s and early 2000s, telecommunication networks and digital imaging technologies became more sophisticated, further expanding the possibilities of teledentistry [17]. High-resolution imaging, electronic health records (EHRs), and the increasing availability of broadband Internet made it easier for dentists and specialists to collaborate remotely [18]. Pilot programs were launched in various countries to test the effectiveness of teledentistry in public health settings, particularly in areas with limited dental infrastructure. These initiatives focused on preventive care, early diagnosis, and patient education, demonstrating the potential of digital tools to bridge gaps in oral healthcare accessibility [19,20].

**Figure 1:**
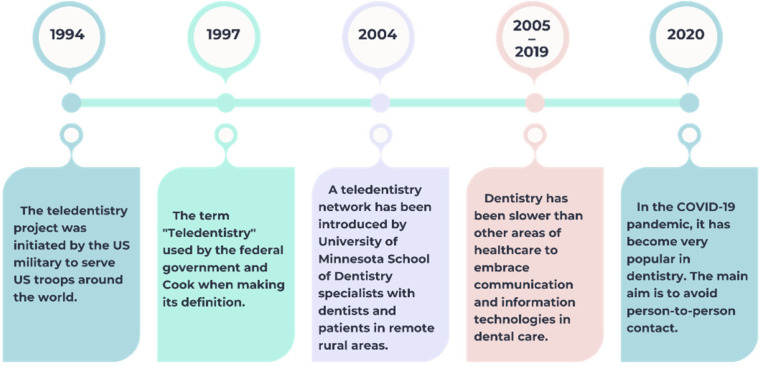
Evolution of teledentistry.

The development of teledentistry has followed a gradual trajectory, beginning with its early application in military settings in 1994, where it served as a tool to provide dental support to U.S. troops stationed abroad [11]. By 1997, the term “teledentistry” was officially adopted in policy and academic circles, laying the foundation for its formal definition [3,6]. The early 2000s brought the first practical implementations, such as the pilot network launched by the University of Minnesota to connect rural patients with dental specialists [14,15,18]. Despite this progress, dentistry remained behind other medical disciplines in adopting telecommunication technologies between 2005 and 2019. A significant shift occurred during the COVID-19 pandemic, when the need to reduce physical contact greatly accelerated the use of teledentistry in both public and private practice [3]. This historical progression—from experimental use to widespread necessity—is visually summarized in Figure 1 below.

### 2.2. The Present

Teledentistry has evolved from an experimental concept into a well-established practice that is transforming modern oral healthcare. Advances in digital technology, artificial intelligence, cloud-based data management, and real-time communication platforms have made remote dental care more accessible, efficient, and effective [21].

One of the key aspects of modern teledentistry is real-time virtual consultations, where patients can interact with dentists through video conferencing platforms. This approach is particularly beneficial for individuals in remote areas, elderly patients with mobility issues, and those who require follow-up consultations without the need for in-person visits [22]. Through these appointments, dentists can assess symptoms, provide treatment recommendations and determine whether an in-office visit is necessary.

Another major advancement in teledentistry is the use of AI-powered diagnostic tools [23]. AI software can analyze dental images, detect early signs of oral diseases, and assist dentists in making accurate diagnoses. These systems enhance efficiency by reducing the time needed for image interpretation and minimizing diagnostic errors [24]. AI-driven chatbots and virtual assistants also provide patients with instant answers to common dental concerns, improving engagement and education [25].

The integration of cloud-based electronic health records has further revolutionized teledentistry. Dentists and specialists can securely access and share patient records in real time, ensuring seamless collaboration and continuity of care [26]. This is especially valuable in multidisciplinary treatments, where specialists from different fields, such as orthodontics, periodontics, and prosthodontics, can work together remotely to develop comprehensive treatment plans [27].

### 2.3. Elements of Teledentistry

**Figure 2:**
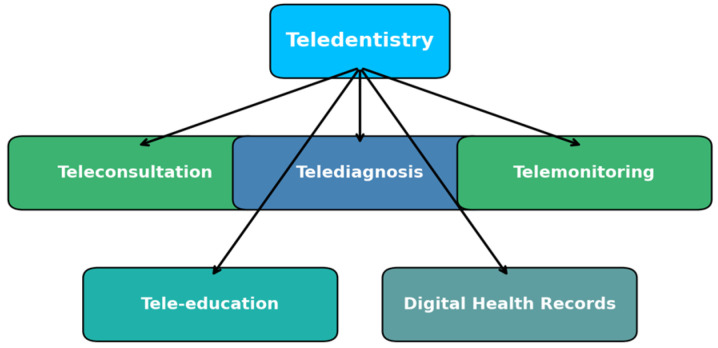
Elements of teledentistry.

Teledentistry is built on several essential components that enable remote dental care. These components integrate various digital technologies to improve accessibility, efficiency, and quality of dental service. The main elements of teledentistry include teleconsultation, telediagnosis, telemonitoring, tele-education, and digital health records, as illustrated in Figure 2 [28].

#### 2.3.1. Teleconsultation

There are two methods that may be utilized for teleconsultation in the field of teledentistry, namely the “Real-Time Consultation” method and the “Store-and-Forward Method” [29]. The real-time consultation process is carried out through the use of a video conference, which allows dental experts and their patients, who are located in various regions, to see, hear, and converse with one another [30].

**Figure 3:**
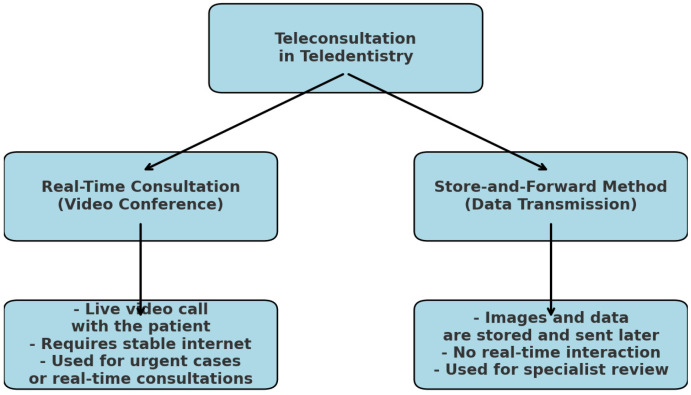
Methods for teleconsultation in dentistry.

The second one comprises the interchange of clinical information and static photographs that have been gathered and saved by the dental practitioner [31]. The practitioner then sends these images to the patient for consultation and treatment planning. Both methods are represented in Figure 3.

The patient is not present while the “consultation” is being conducted. Information on patients, radiographs, graphical representations of periodontal and hard tissues, treatments that have been administered, laboratory findings, tests, notes, images, and other information that may be sent across different providers can be shared by dentists. Patients, particularly those who require the consultation of a specialist, may find this data exchange to be of the utmost interest [32,33].

The use of unprotected email for communication between patients and healthcare providers has been the subject of research in the United States of America since the early 1990s [34,35]. The use of email in patient–provider contact has been the subject of a number of research [36,37] since this momentous event. The year 2004 saw the publication of a study that provided a comprehensive overview of the current state of electronic patient record systems and their use in healthcare in general [38]. However, concerns regarding patient privacy, data security, and compliance with healthcare regulations have been central to discussions on the risks and limitations of using unencrypted email in medical settings [39]. On the other hand, there is a fairly limited number of studies of this kind relating to dentistry [40,41].

Early research in United States focused on evaluating the benefits of email communication for non-urgent medical inquires, prescription refills, and appointment scheduling [42]. Studies indicated that email could reduce the burden on in-person visits, improve patient engagement and streamline healthcare processes [43,44,45]. However, these advantages were often outweighed by concerns related to data breaches, unauthorized access and lack of encryption. Email systems used in the early years of digital communication lacked built-in security features, making protected health information vulnerable to interception [46].

Legal and ethical issues surrounding unprotected email communication gained attention following the introduction of the Health Insurance Portability and Accountability Act in 1996, which set strict guidelines for the protection of electronic health information in U.S. [47]. Subsequent research emphasized the need for secure communication channels, such as encrypted messaging platforms and secure patient portals, to comply with data protection laws [48]. There are no nationally ratified regulations for the use of email in patient–provider communications or consultation.

On the other hand, despite the fact that a sizeable number of dentists appear to make use of some kind of electronic communication tool on a regular basis for the purpose of consultation or patient communication, there is a dearth of knowledge regarding how to make productive and secure use of electronic communication channels [49]. As a result of this, dentists should receive further training in this area as part of their specialty training program, and, in the future, additional training in this area should also be included in basic dental training [50].

It has also been reported that there is a third method, known as the “Remote Monitoring Method”. In this approach, patients are observed from a distance and the monitoring can take place either in a hospital or at home [51].

In public health, teledentistry has played a crucial role in expanding access to underserved populations [52]. Governments and healthcare organizations have implemented teledentistry programs in schools, rural areas, and developing countries where dental services are scarce [53]. Mobile dental units equipped with digital imaging and Internet connectivity allow remote diagnosis and treatment planning, ensuring that more people receive essential oral healthcare [54].

#### 2.3.2. Telediagnosis

Telediagnosis is a pivotal aspect of teledentistry, referring to the remote diagnosis of dental conditions using telecommunications technology [55]. This approach enables dental professionals to assess and diagnose patients without the need for in-person consultation, thereby enhancing accessibility and efficiency in dental care [56].

An investigation of the function of telediagnosis in oral medicine was carried out by Cardozo and colleagues [57]. Through the use of email, the clinical information of sixty patients was transmitted in the form of photographs [57]. In the field of oral medicine, the utilization of information technology has the potential to improve the precision of consultations. As was to be predicted, the presence of two specialists who were located remotely enhanced the likelihood of making an accurate diagnosis [57].

##### Teledentistry in Orthodontics

The notion of teleorthodontics is a direct reflection of the growing need for and expansion in this sector [58]. In this age of technological globalization, the globe is becoming smaller in terms of connectivity. In addition to enhancing communication and contact between patients and healthcare professionals, teleorthodontics also allows for improved communication and interaction between doctors [59,60].

Lately, there has been a rise in the number of patients looking for orthodontic treatments that do not need as many office visits while still letting the specialist monitor their treatment progress [61]. Some have speculated that the recent uptick in the popularity of teleorthodontics is due to the fact that it allows patients to keep control over their treatment while reducing the number of people needlessly visiting orthodontic offices [62].

Due to its simplicity, convenience, and positive association, video consultations were deemed the most patient satisfying in prior research [63]. Researchers found that patients who used teleorthodontics were more likely to follow their prescribed oral hygiene routines, and that the technology was easy to use and convenient for patients. Thanks to advancements in communication technology, teleorthodontics consultations may now take place remotely, saving patients the trouble of traveling to and from appointments [64].

The COVID-19 epidemic intensified the demand for teleorthodontics services to address orthodontic crises. Research by Arqub et al. [65] has shown that in cases of orthodontic emergencies, the majority of patients (73%), rather than visiting the office, preferred to be contacted by phone or text. By categorizing orthodontic emergencies into three groups—loose brackets, probing ligatures and far wires, and damaged retainers and aligners—Caprioglio et al. [66] suggested standards for the management of orthodontic emergencies using telediagnosis in orthodontics.

In a recent systematic review, Rouanet et al. [67] also investigated the usefulness of teleorthodontics as a tool; the review included a large number of research designs to probe the subject thoroughly. They summed up the pros and cons of teleorthodontics based on the findings of their systematic review. The benefits include resolving orthodontic emergencies, anticipating the next appointment (especially in an emergency), better retention appointment follow-up, and easier communication between patients and practitioners [67]. Conversely, the following are some drawbacks that were identified: full-remote treatment poses risks that should be avoided; not all orthodontic treatments can be completed remotely; there are concerns about privacy, safety, and data protection [67].

##### Teledentistry in Detecting Dental Caries

Telediagnosis also plays a crucial role in the early detection of dental caries by leveraging digital imaging and remote consultation [68]. Through advanced imaging techniques, such as intraoral cameras, digital radiographs, and AI-based analysis, dentists can assess and diagnose cavities without requiring the patient to visit the clinic physically [69]. This approach enhances access to dental care, particularly for individuals in remote areas, and facilitates timely intervention, ultimately improving oral health outcomes.

By utilizing a mail-based system, Torres-Pereira et al. [70] were able to remotely diagnose oral lesions by applying the concepts of teledentistry. The study’s findings proved that dental deformities may be accurately diagnosed remotely.

Research conducted by Amável et al. involved Portuguese kindergarteners ranging in age from four to six [71]. To diagnose dental cavities, he employed a teledentistry model based on store-and-forward photography. The photographic approach demonstrated very high levels of sensitivity (from 94% to 100%) and specificity (52% to 100%), according to the results [71].

##### Teledentistry in Endodontics

Teledentistry allows for the delivery of endodontic therapy to low-income individuals. Since root canal orifices can be remotely recognized using teledentistry, it stands to reason that general dentists can benefit from the guidance and instruction of highly trained endodontists when it comes to root canal orifice recognition [72]. Diagnosing periapical lesions of the front teeth have been successfully accomplished with the use of teledentistry, a kind of online contact [73]. In addition to making emergency aid more accessible, it also reduces the expenditure of long-distance trips.

##### Teledentistry in Implantology and Oral Surgery

Prior studies have shown that teledentistry is a viable option for maxillofacial surgery consultations, evaluations, treatment planning, and follow-up care [74,75,76,77]. Compared to on-site evaluations, teledentistry, which involves dental surgeons and patients meeting over the phone for various types of maxillofacial surgery procedures, was well-received [78].

Although teledentistry cannot replace the hands-on nature of surgical procedures, it has found a supportive role in preoperative consultations, treatment planning, and postoperative follow-ups. In implantology, virtual case evaluations and 3D imaging tools enable clinicians to remotely assess bone availability, prosthetic space, and patient eligibility. Teledentistry platforms can be used to coordinate multidisciplinary cases and share surgical guides. Clinical diagnosis of impacted or semi-impacted third molars helped by telemedicine was shown to be equivalent to real-time clinical diagnosis, according to Duka M et al. [79].

Telemedicine consultations are just as reliable as traditional methods when it comes to evaluating patients for dentoalveolar surgery with general anesthesia and nasotracheal intubation. Telecommunication is a cost-effective and efficient way to provide preoperative evaluations in cases where patient transport is difficult or expensive, according to Masongo et al. [80].

For oral surgery, remote monitoring of postoperative healing, suture assessment, and patient-reported outcomes can reduce the need for in-person visits and improve continuity of care.

Telediagnosis in dentistry is a transformative approach that enhances accessibility, efficiency, and accuracy in dental care. Additionally, telediagnosis supports early detection, leading to improved treatment outcomes and preventive care.

Smartphones, according to Aziz et al., enable oral and maxillofacial surgeons to move around freely without being limited by a desktop computer, and they also give easy access to digital pictures sent via email. This leads to better triaging and a more efficient specialty consultation, which benefits the maxillofacial patient in the long run [81].

As technology continues to advance, integrating telediagnosis into routine dental practice will further enhance patient care and streamline diagnostic processes in modern dentistry [82].

##### Teledentistry in Pediatric Dentistry

Early identification, education, and preventative care are three areas where teledentistry is finding increasing use in pediatric dentistry. Pediatric dentists can evaluate eruptive patterns, caries risk, oral habits (such thumb sucking or bruxism), and offer remote behavioral coaching for worried youngsters through virtual consultations [83]. In order to assist prevent unnecessary clinic visits, dentists may now use video-based assessments to advise parents on urgent symptoms and determine if an in-person appointment is essential [84].

Children and their carers can benefit from interactive oral health education through teledentistry, which increases the likelihood that they will follow the recommended brushing and eating habits [84]. Because of the potential lack of consistent access to pediatric dental specialists, this is especially helpful in school-based programs and underprivileged neighborhoods.

#### 2.3.3. Telemonitoring

Using digital equipment and communication technology, telemonitoring allows for the continuous remote surveillance of patients’ oral health, which is a novel method in dentistry. Without the need for regular in-office visits, dentists may evaluate treatment progress, post-surgical recuperation, and offer individualized suggestions [85].

New ways of communicating and a more demanding attitude towards the rapid fulfilment of information needs and help requests have emerged with the growing distribution and technical advancements in computers, cellphones, and tablets, particularly among teenagers [86].

Opportunities in teledentistry include cutting-edge technology, such as kiosks, apps for monitoring websites, mobile wearables, and video conferencing [87]. Several studies have already verified the use of various text messaging apps, like WhatsApp, Telegram, or WeChat, as teledentistry opportunities [88,89], particularly when patients wish to communicate with clinicians for emergencies [90] or to promote good oral health [91], which is not surprising given that most people own a smartphone [92].

Orthodontic treatments, periodontal disease management, and postoperative care are areas where this system exceeds [93]. Especially for patients in underserved or far-flung locations, telemonitoring increases efficiency, improves preventive treatment, and broadens access to high-quality dental services [94].

Dental monitoring (DM, Dental Monitoring©, Montreal, France) [95] is an advancement in orthodontic software that enables the therapist to stay updated using the patient’s regular picture taking in order to determine appliance integrity and the effectiveness of oral hygiene practices while the patient undergoes orthodontic treatment.

Patients appreciate the convenience of remote monitoring, especially those with mobility issues or who live in remote areas. However, some express concerns about the accuracy of remote assessments compared to in-person visits [96]. Research by Sharif et al. [97], showed that the majority of patients were unaware that telemonitoring was an option. Telemonitoring, according to some, does not constitute cutting-edge, high-quality care. They likely do not define high-quality treatment based on the amount of technology their orthodontist offers, which is why this negative comment makes sense to them [97]. If it produces desirable outcomes, even a traditional treatment seems to be of a high level. The application’s potential for dental calculation may have been undervalued because of the belief that telemonitoring did not constitute a high-quality technology [98].

#### 2.3.4. Tele-Education

Tele-education in dentistry is an innovative approach that enhances learning opportunities for dental students, practitioners, and patients by using digital platforms and communication technologies [99]. It provides access to high-quality educational content, live lectures, recorded sessions, and interactive case discussions, eliminating geographical barriers. With advancements in e-learning tools, virtual simulations, and artificial intelligence, tele-education has become a vital component of modern dental training [100].

One of the key benefits of tele-education is its ability to connect students and professionals with experts worldwide. Webinars, online courses, and virtual reality-based training enable real-time learning and skill development without requiring physical presence [101]. This is especially valuable for continuing education, allowing dentists to stay updated with the latest research, techniques, and technologies.

For patients, tele-education plays a crucial role in promoting oral health awareness. Digital resources, such as instructional videos, mobile apps, and online consultations help educate individuals about preventive dental care, treatment options, and post-treatment guidelines. This empowerment leads to better oral health practices and improved patient compliance [102].

In underprivileged or vulnerable communities, teledentistry has the ability to greatly improve health literacy [100]. Personalized explanations of diagnoses, demonstrations of proper oral hygiene procedures, and clarification of treatment plans are all made possible by teledentistry’s user-friendly platforms that allow direct connection between patients and practitioners [15,16]. As a result, patients are better able to comprehend their care and follow doctors’ orders.

Even those with little formal education or limited reading abilities may understand complicated dentistry topics with the help of digital platforms that include visual aids, multilingual information, and interactive courses [15]. Since better health outcomes and lower disease burdens have been associated with higher levels of general and oral health literacy, this is particularly important in public health settings [1,2].

In addition to facilitating queries and reviews of instructions through recorded materials, teledentistry gives patients access to credible educational information outside of clinical contact, which promotes lifelong learning. This promotes independence and active participation in one’s own health by changing the paradigm from reactive to proactive approaches to dental health management [5,9].

Despite its advantages, tele-education also faces challenges, such as limited hands-on experience, technological accessibility issues, and the need for proper accreditation of online programs. However, with continuous improvements in digital learning, tele-education is expected to become an indispensable part of dental education, ensuring wider access to knowledge and training [103].

#### 2.3.5. Digital Health Records

Digital records in dentistry have revolutionized the way that patient information is collected, stored, and accessed. Unlike traditional paper-based records, electronic dental records (EDRs) provide a more efficient, secure, and organized way to manage patient data [104]. These records include medical histories, diagnostic images, treatment plans, prescriptions, and progress notes, allowing for seamless documentation and easy retrieval.

Computers are used extensively at dental offices to assist with patient appointments and invoicing, according to a survey of dental offices conducted amongst people across the country [105]. In addition, dental offices are gradually installing computer access in patient waiting areas. This could help with patient check-in, give patients another place to obtain educational materials, and enable future updates to their dental, medical, and medication records through a patient portal [106,107].

Dentists are increasingly using chairside computers to access the Internet. This allows them to access patient education materials, information on goods and vendors, and evidence-based data to help with clinical decision-making [108].

Additionally, digital records enhance patient safety by minimizing errors related to manual documentation and lost files. Cloud-based storage solutions ensure that data is securely backed up and can be accessed remotely when needed. Patients also benefit from improved communication, as digital records can be shared easily between providers, ensuring continuity of care [109].

### 2.4. Dentist–Patient Relationships in Teledentistry

Mutual trust is essential in the dentist–patient relationship for the patient to comply with the dentist’s requests for information and for the patient to accept the dentist’s treatment plan. As such, it is important to do this when both parties have agreed upon it. For online consultations to be effective, the agreement should lay forth certain ground rules.

As a result, the dentist needs to have the patient’s full approval before making any judgements, such as whether or not online dental care is acceptable [110]. Because of this, patients and dentists should only engage in online dental treatment when they have an established rapport. In a suitable situation, in-person and online dental treatment must be provided by the same dentist.

The primary role of dentists in teledentistry is to provide online consultations. Because of this, the dentist takes great care in determining if enough information is gleaned from the virtual consultation for a proper diagnosis. A dentist’s main responsibilities during a teledentistry consultation include making a diagnosis, coming up with a treatment plan, finding out what basic and preventative oral health services patients in their community could receive, and speeding up referrals and follow-ups when needed [111].

**Figure 4:**
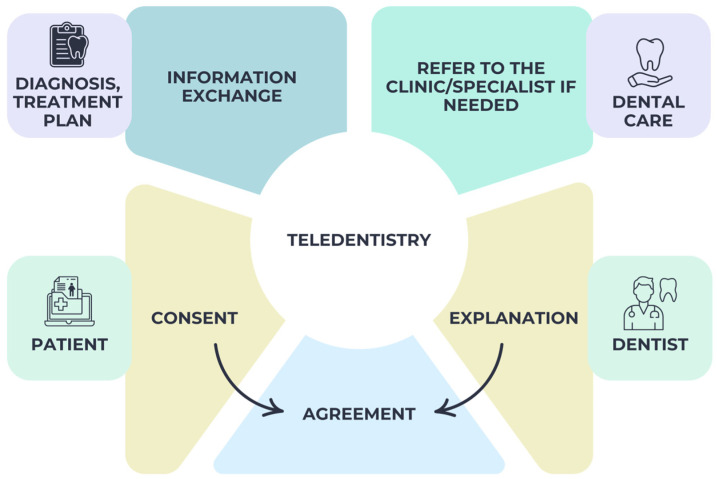
Doctor–patient relationship in teledentistry.

Furthermore, dentists ensure the safety of their patients’ information by using adequate security measures for both the transmission and storage of patient data. When making a diagnosis and planning treatment, a dentist should think about the patient’s goals, the likelihood of success, any complications that may arise, and any unanswered questions or concerns that may have persisted after the initial appointment [112]. As a result of all the above, Figure 4 illustrates the relationship that exists in teledentistry between the doctor and the patient.

It is a cornerstone of the dentist–patient relationship that the patient’s confidence that the dentist will keep their information confidential will always be upheld. While patients should be able to keep their health information private, dentists need this information in order to offer safe treatment to patients. Important privacy information that patients should be able to access includes who can access their medical records, with whom those records are shared, the reasons for any third-party sharing of medical records, where they can request copies of their personal records, and what information, if any, is obtained about them [113,114].

Several recent studies have examined the effectiveness of teledentistry in improving patient outcomes [115]. One of the most significant benefits is enhanced access to timely dental care, particularly in underserved or rural populations. By reducing travel time and offering flexible appointment options, teledentistry increases the likelihood of early detection and intervention, especially in conditions, such as dental caries, soft tissue lesions, or postoperative complications [56].

Patient satisfaction with teledentistry services is generally high, with many reporting increased convenience, reduced anxiety, and greater involvement in their care decisions. In chronic conditions, like periodontal disease or post-orthodontic maintenance, teledentistry supports continuous monitoring, which helps improve treatment adherence and outcomes [110]. Moreover, remote education and motivational interviewing via digital platforms have been shown to improve oral hygiene behavior over time.

However, limitations remain in evaluating long-term outcomes due to the relative novelty of widespread teledentistry adoption. More randomized controlled trials and longitudinal studies are needed to quantify its impact on clinical endpoints, such as tooth retention, disease recurrence, or cost-effectiveness.

These are all typical enquiries that a reliable teledentistry supplier should be able to address. Consequently, a teledentist’s practice is subject to the same regulations governing patient confidentiality as any other medical professional. When using teledentistry, dentists should be very careful to protect their system and the information they provide [116].

For this part to work, the patient must be open to the idea of teledentistry. Anxieties could arise if the patient thinks they will not be able to ask their dentists all their questions or have their concerns addressed in person [117]. Teledentistry is becoming increasingly popular among patients as telemedicine continues to grow. Both patients and dentists are starting to take notice of teledentistry, according to a slew of research [118].

There are a lot of moving parts in teledentistry, and most dentists throughout the world may find it difficult to learn and implement [119,120]. Their fears of making a wrong diagnosis compound their difficulty with the technological aspects of the operation. For some patients, dentists are also worried about the increased degree of cause and cost. The field is constrained by insufficient training facilities, unsuitable technical gadgets and assistance, and slow Internet connections [121].

Dentists also face a number of obstacles, including inadequate guidelines, low reimbursement rates, a lack of coordination, disconnected central and remote centers, expensive infrastructure, and an inharmonious relationship between teledentistry organizations and healthcare service providers [122]. The inability to assess pulp vitality and tooth percussion is still another limit.

It is essential to resolve the aforementioned issues in order to accept the growth of teledentistry. Consequently, it is imperative that dentists receive enough training and continuously enhance their understanding of technology.

### 2.5. Global Utilization and Regional Disparities in Teledentistry

In the ever-changing healthcare landscape, teledentistry stands out as a promising solution to expand access to dental treatment. As a result, dental treatment is becoming accessible to formerly inaccessible groups, despite physical distance [10,26]. It is enabling prompt consultations, follow-ups, and preventive care, seeking to improve oral health outcomes [3,4].

The regulatory climate, healthcare financing, digital literacy, and technical infrastructure of a nation are the primary determinants of the rate of teledentistry adoption in that country [13,14,15]. Following the COVID-19 pandemic, teledentistry was more extensively integrated into public and commercial healthcare models in high-income nations, such as the US, Canada, Australia, and EU members [22,26,33]. These areas have favorable conditions for virtual treatment, including the widespread availability of high-speed Internet, EHR systems, and payment policies [28,29,30,31].

On the other hand, there are usually substantial implementation hurdles for low- and middle-income nations. Some of these issues include a dearth of national telehealth legislation, a small number of clinicians per 100,000 people, and slow Internet speeds [31,40]. Nevertheless, teledentistry for screening and triage has been used in some pilot projects with the help of mobile devices and community health professionals. These programs have been especially effective in rural or isolated locations [23,27,36].

As global health policies increasingly recognize oral health as integral to general health, international collaboration and support for digital dental tools in developing regions may help reduce inequities in oral healthcare access [36,50].

Teledentistry supports the healthcare system by reducing the burden on emergency dental services, optimizing appointment scheduling, and minimizing unnecessary clinic visits, particularly in high-demand urban centers or geographically isolated regions. By enabling remote triage and follow-up, it helps allocate resources more efficiently, allowing in-person care to be reserved for urgent or complex procedures [52].

In public health contexts, teledentistry enhances preventive care outreach, supports interdisciplinary collaboration, and facilitates early identification of oral–systemic conditions, thereby aligning dental care with broader health strategies. Overall, it strengthens health system resilience by integrating oral health into digital health transformation efforts [52,57,64].

## 3. Future Perspectives

Despite teledentistry’s existence since the 1990s, the global pandemic scenario has only recently brought its relevance to light. Given that the pandemic is far from over, teledentistry is expected to endure for quite some time [122]. Teledentistry is set to transform the future of oral healthcare by integrating new technologies, expanding accessibility, and improving the efficiency of dental services. As the world moves toward more digital healthcare solutions, several advancements will shape the next generation of teledentistry [123].

The development of teledentistry has made it possible for patients who are housebound or otherwise unable to leave their homes to have dental consultations and treatments as easily as pressing a phone button.

On the other hand, teledentistry applications in the elderly have received little investigation [124]. Therefore, in addition to particular ethical and legal requirements, there are still a number of things that need to be addressed about teledentistry, including techniques, efficacy, dependability, indications (oral and dental needs), applications (dental services), and, most importantly, the attitudes and interests of senior dental patients [124].

It is reasonable to have an optimistic outlook on the now-accelerated sustainable development of teledentistry. Patients and doctors alike stand to gain much from the expansion of digital service options, which can improve many facets of daily life. One such area is the accessibility of dentistry and other medical care.

A solid foundation for building teledentistry services is the active engagement and collaboration of dental workers, with an acceptance rate of up to 61%. Because teledentistry is a relatively new technology that is not grounded in sound evidence-based medicine, it may not be well-received by dental professionals and might have serious negative consequences when used for diagnosis and treatment [123,125].

Despite the promising advantages of teledentistry, several limitations hinder its widespread implementation and effectiveness. A significant concern is the lack of standardized clinical protocols, which affects the consistency and reliability of remote diagnosis and treatment planning [126].

Data security and patient confidentiality remain critical challenges, particularly in systems that do not comply with established privacy regulations, such as GDPR or HIPAA. Moreover, technological disparities between urban and rural areas can lead to unequal access to teledental services, exacerbating existing health inequities [127,128].

Additionally, insufficient digital literacy among both patients and practitioners can limit the effectiveness of virtual consultations. Dentists often face a steep learning curve when adopting new technologies and may require additional training to navigate digital tools confidently. Legal and reimbursement barriers also pose difficulties, as not all regions provide clear guidelines or financial support for teledentistry [129].

Although there are a number of ethical and clinical restrictions surrounding teledentistry, it can provide potential answers for remote access and monitoring. The lack of in-person contact and the possibility of misunderstandings makes the process of acquiring informed consent a major cause for worry when it comes to virtual settings [130]. Patients need clear digital permission forms and explanations of the limitations and hazards of remote evaluations in order to understand them.

Another difficulty arises when dealing with emergencies, since teledentistry might not be enough. Acute infections, injuries, or uncontrolled bleeding are examples of situations where rapid clinical attention is necessary, and remote consultation cannot be a substitute for it [131]. The only way to lessen the impact of this danger is to have well-defined triage procedures and to work in tandem witmh local emergency services.

Special needs patients, including those with speech difficulties, cognitive disabilities, or mental health disorders, can also pose challenges to teledentistry treatment [132]. Patients with these conditions may have trouble communicating their symptoms, understanding and following directions, or interacting with digital tools. Personal interaction may continue to be crucial for these people [133]. Addressing these gaps and ensuring fair service delivery may be achieved via the use of adaptive communication technologies, carer support, and hybrid care models.

Despite its growing relevance, teledentistry has inherent limitations and cannot fully replace in-person clinical practice, especially in situations that require physical examination, tactile feedback, or operative interventions. Procedures, such as dental extractions, restorations, root canal therapies, and implant placements, are impossible to perform remotely and demand the direct presence of a clinician.

Furthermore, patients with dental anxiety, language barriers, or complex health histories may benefit more from face-to-face interaction, where the provider can offer reassurance, interpret non-verbal cues, and adapt the communication dynamically.

These challenges underline the importance of further research, policy development, and investment in infrastructure to ensure the safe, ethical, and equitable integration of teledentistry into routine dental care.

Nevertheless, the accelerated adoption of digital healthcare services provides an opportunity for sustainable development in the dental field. By addressing existing challenges and refining teleconsultation methods, teledentistry has the potential to bridge the gaps in dental care accessibility while complementing traditional in-person treatment.

## 4. Conclusions

Teledentistry has emerged as a transformative solution in modern oral healthcare, offering improved accessibility, efficiency, and patient engagement. Its integration into various dental specialties, such as orthodontics, periodontics, and pediatric dentistry, has enhanced remote consultations, digital diagnostics, and treatment planning.

The future of teledentistry appears promising as technology advances, enabling the development of smarter diagnostic tools, improved telecommunication systems, and enhanced digital health records. With proper regulation, training, and integration into dental practice, teledentistry has the potential to complement traditional dental care, making oral health services more inclusive and accessible, especially for remote and underserved populations.

Ultimately, while there are limitations to address, the continuous evolution of digital healthcare services ensures that teledentistry will play an increasingly significant role in modern dentistry.

## Abbreviations

The following abbreviations are used in this manuscript:

TDA	Total Dental Access
AI	artificial intelligence
U.S.	United States
DM	dental monitoring
EDRs	electronic dental records
